# Supplementary material for: Liquid-shaped microlens for scalable production of ultrahigh-resolution optical coherence tomography microendoscope
Source: Commun Eng. 2024 Jan 2;3:1. doi: 10.1038/s44172-023-00157-1 (PMC10955823; doi:10.1038/s44172-023-00157-1)
Supplement: Supplementary file 2 — Supplementary Information [file 44172_2023_157_MOESM2_ESM.pdf]

Supplementary Information for Liquid-shaped microlens for scalable production of ultrahigh-resolution optical coherence tomography microendoscope

Chao Xu[1], Xin Guan[2], Syeda Aimen Abbasi[1], Neng Xia[3], To Ngai[2], Li Zhang[3], Ho-Pui Ho[1], Sze Hang Calvin Ng[4], and Wu Yuan[1,*]

[1]Department of Biomedical Engineering, The Chinese University of Hong Kong, Hong Kong SAR, China
[2]Department of Chemistry, The Chinese University of Hong Kong, Hong Kong SAR, China
[3]Department of Mechanical and Automation Engineering, The Chinese University of Hong Kong, Hong Kong SAR, China
[4]Department of Surgery, The Chinese University of Hong Kong, Hong Kong SAR, China
*wyuan@cuhk.edu.hk

The PDF file includes:

Supplementary Notes 1-4

Figs. S1 to S8

Supplementary Tables 1-6

Other Supplementary Materials available for this manuscript include the following:

Movies S1-2

Contents

Supplementary Information for Liquid-shaped microlens for scalable production of ultrahigh-resolution optical coherence tomography microendoscope 1

Supplementary Note 1: Shrinkage of microlenses before and after polymerization.................. 3

Supplementary Note 2: Characterization and imaging results of simultaneously fabricated liquid-shaped microendoscopes. 4

Supplementary Note 3: Ultrahigh-resolution endoscopic SD-OCT system............................... 5

Supplementary Note 4: Choice of optical liquids for microlens fabrication.............................. 6

Figure S1. Procedures for fabricating liquid-shaped microlens and microendoscope. 7

Figure S2. Imaging results of simultaneously fabricated microendoscopes. 8

Figure S3. Schematic of the endoscopic spectral-domain OCT (SD-OCT) system working near 800 nm. 9

Figure S4. Illustration of OCT scanning in mouse deep brain and the *en face* projection procedures. 10

Figure S5. Long-term stability on imaging performance of fabricated microendoscopes. 11

Figure S6. Calibration of dispensed liquid volume. 12

Figure S7. Refractive index profile of NOA 81 under room temperature. 13

Figure S8. Schematic of mouse brain handling for histology. 14

Supplementary Table 1: Volume variation of microlenses before and after polymerization. .. 15

Supplementary Table 2: Characterization of the simultaneously fabricated liquid-shaped microendoscopes. 16

Supplementary Table 3: Comparison of fabrication methods for OCT microendoscopes. 17

Supplementary Table 4: Six groups of glass substrates with different parameters of surface wettability modification. 18

Supplementary Table 5: Two groups of cylinder substrates with different parameters of surface wettability modification. 19

Supplementary Table 6: Comparison between optical liquids. 20

Movie S1: Microlens on a circular cylinder substrate... 21

Movie S2: Microlens on an elliptical cylinder substrate. 21

Supplementary Note 1: Shrinkage of microlenses before and after polymerization.

There is a scale-invariable shrinkage of the liquid-shaped micro-lens after the complete polymerization, which keeps the contact angle and shape unchanged. To study the shrinkage ratio of NOA 81-based micro-lens, a 365-nm ultraviolet lamp was used to provide a power density of 65 mW/cm^2 (on lens sample). The volumes of the micro-lens before (V_1) and after (V_2) polymerization were measured using a liquid drop analysis system (OCA 25, Dataphysics Instruments GmbH) at room temperature of 25°C. As shown in Supplementary Table 1, the average shrinkage ratio in volume (defined as $\frac{V_1 - V_2}{V_1}$) is found to be about 7.69% with a standard deviation of 0.39% for NOA 81-based micro-lens.

Therefore, an increase of 8.33% of the calculated volume of the designed microlens is used to compensate for the shrinkage effect to help achieve the desired shape and size of the polymerized microlens. As for the microlens used in microendoscopes, the dispensed liquid volume was about 6.2 nL to achieve a semi-spherical lens of a radius of 140 μm and lens volume of about 5.7 nL.

Supplementary Note 2: Characterization and imaging results of simultaneously fabricated liquid-shaped microendoscopes.

Five microendoscopes were simultaneously fabricated, including two rigid ones (# 1 and # 2) and three flexible ones (# 3, # 4, and # 5). They were characterized of similar results (see Supplementary Table 2) and tested for OCT imaging (Fig. S2) with comparable image qualities, showing the scalability of the liquid shaping technique for microendoscope fabrication.

Supplementary Note 3: Ultrahigh-resolution endoscopic SD-OCT system.

To characterize the performance of the microendoscope, a homemade endoscopic spectral-domain OCT (SD-OCT) system was built (Fig. S3). The custom SD-OCT system employed a superluminescent diode source (MT-850-HP, Superlum Inc.) with a 3-dB bandwidth of 160 nm and a central wavelength of about 842 nm. A 50:50 fiber coupler (TW850R5A2, Thorlabs Inc.) was adopted to split the light into the reference and sample arms. The light beam in the reference arm was collimated using a reflective collimator (RC02APC-P01, Thorlabs Inc.). A pair of N-SF11 prisms (Edmund Inc.) were inserted between the reflective collimator and the reflective mirror (PF05-03-P01, Thorlabs Inc.) to compensate for the dispersion imbalance between the reference and sample arms. A homemade rotary joint was mounted on a linear translational stage (X-LSM150A, Zaber Technologies) to rotate and pull back the imaging probe and thus obtain three-dimensional volumetric images. The OCT light back-reflected from reference and sample arms interferences with each other and is collected using a spectrometer (Cobra-S 800, Wasatch Photonics Inc.) with a high line-scan rate up to 250 kHz. To manage the polarization mode dispersion (PMD), two polarization controllers (FPC030, Thorlabs Inc.) are utilized. The imaging depth of the system is about 1.04 mm.

Supplementary Note 4: Choice of optical liquids for microlens fabrication.

Six representative optical liquids, including NOA 60, NOA 61, NOA 63, NOA 65, NOA 68, and NOA 81 (Norland Products Inc.), have been considered to fabricate the microlens (see Supplementary Table 6). NOA 81 is selected because of (1) fast curing property (20 minutes UV light exposure at a power density of 65 mW/cm^2 for complete polymerization); (2) good endurance against temperature variation (withstand -150~125°C after polymerization); (3) its low viscosity, which makes it easy to dispense; (4) relatively high elastic modulus, which makes it tough and not easy to deform; and (5) relatively high transmission near 800 nm (as high as 96%).

Figure S1. Procedures for fabricating liquid-shaped microlens and microendoscope.

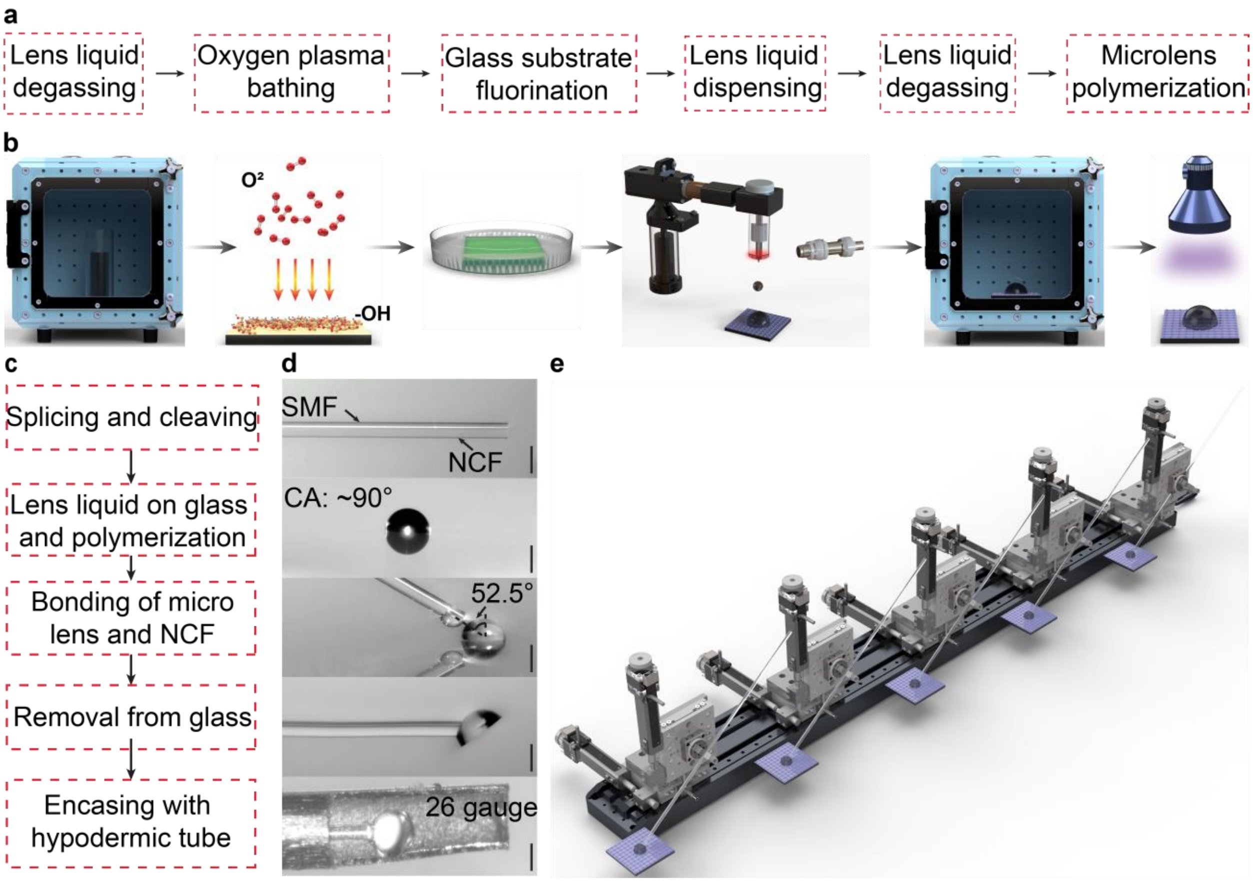

Fig. S1 Procedures for fabricating liquid-shaped microlens and microendoscope. a The flow chart of microlens fabrication. **b** The procedures for fabricating the liquid-shaped microlens. Different colors of the glass substrate indicate the modification process of the glass surface property: yellow indicates that the hydroxy (-OH) was exposed on the glass surface during oxygen plasma cleaning; green indicates that the fluoride was reacting with the hydroxy; purple indicates that the hydroxy was bonded entirely with the fluoride and the glass substrate was fully fluorinated. **c** The flow chart of liquid-shaped microendoscope fabrication. **d** Corresponding photographs of the fabrication procedures listed in **c**. **e** Simultaneous fabrication of the imaging probes. All scale bars are 200 μm.

Figure S2. Imaging results of simultaneously fabricated microendoscopes.

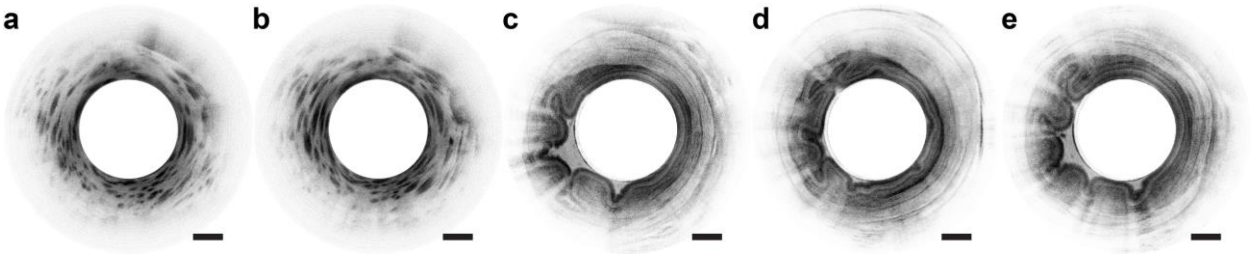

Fig. S2 Imaging results of simultaneously fabricated microendoscopes. From left to right, the first two images (**a** and **b**) show 2D cross-sectional images of mouse brain obtained from # 1 and # 2 rigid microendoscopes, respectively. The last three images (**c**, **d**, and **e**) show the cross-sectional images of rat esophagus obtained from # 3, # 4, and # 5 flexible microendoscopes, respectively. The imaging results demonstrate the comparable performance of the simultaneously fabricated microendoscopes. # 1 and # 3 are the reported rigid and flexible microendoscopes, respectively. All scale bars are 250 μm.

Figure S3. Schematic of the endoscopic spectral-domain OCT (SD-OCT) system working near 800 nm.

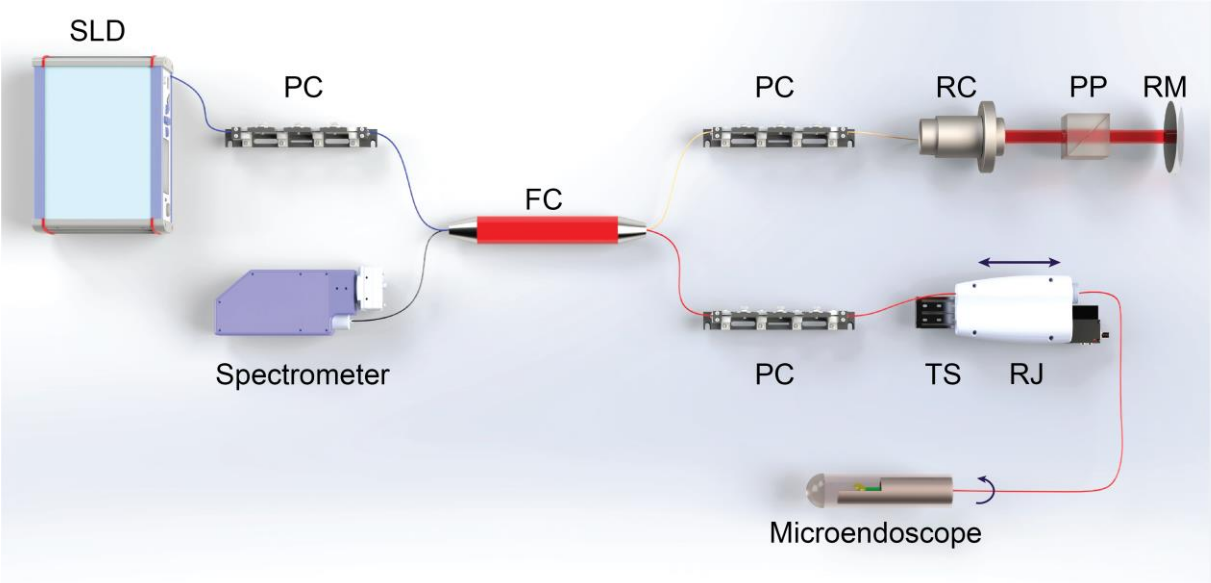

Fig. S3 Schematic of the endoscopic spectral-domain OCT (SD-OCT) system working near 800 nm. SLD: superluminescent diode, PC: polarization controller, FC: fiber coupler, RC: reflective collimator, PP: prism pairs, RM: reflective mirror, TS: translational stage, RJ: rotary joint.

Figure S4. Illustration of OCT scanning in mouse deep brain and the *en face* projection procedures.

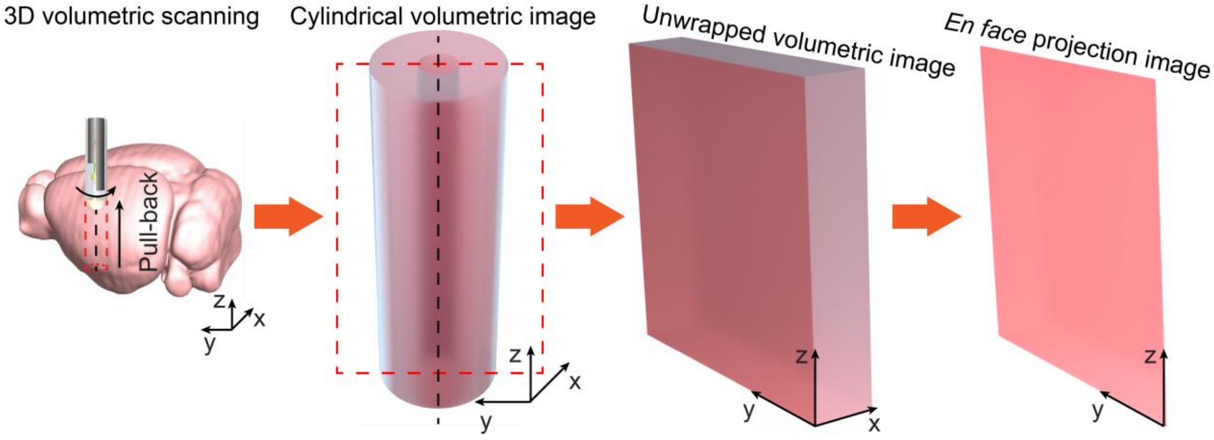

Fig. S4 Illustration of OCT scanning in mouse deep brain and the *en face* projection procedures.

Figure S5. Long-term stability on imaging performance of fabricated microendoscopes.

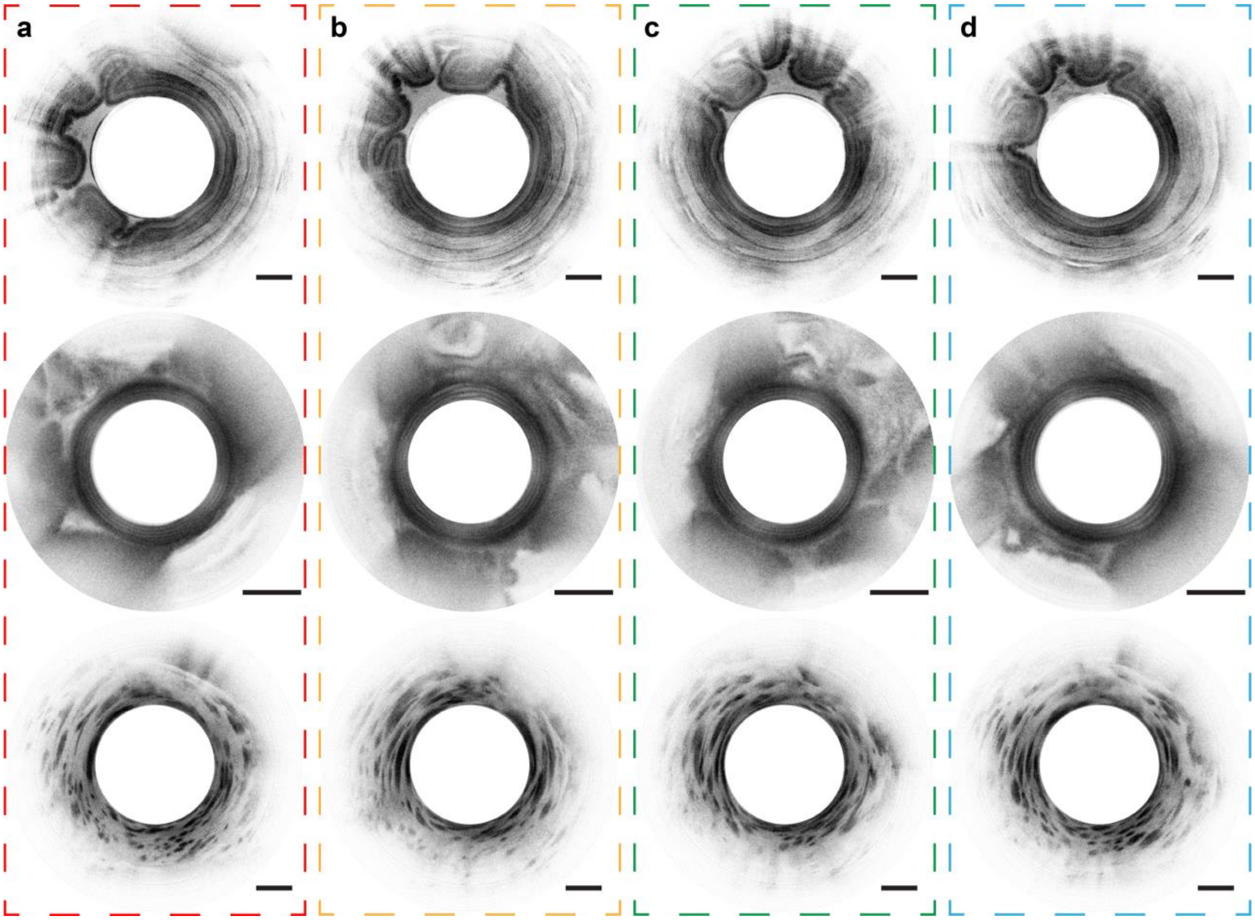

Fig. S5 Long-term stability on imaging performance of fabricated microendoscopes. The 2D cross-sectional images of the rat esophagus, mouse aorta, and mouse deep brain (from top to bottom) obtained when the microendoscopes were fabricated at 1 month (**a**), 3 months (**b**), 6 months (**c**), and 12 months (**d**). The comparable imaging results demonstrate the long-term stability of our microendoscopes. All scale bars are 250 μm.

Figure S6. Calibration of dispensed liquid volume.

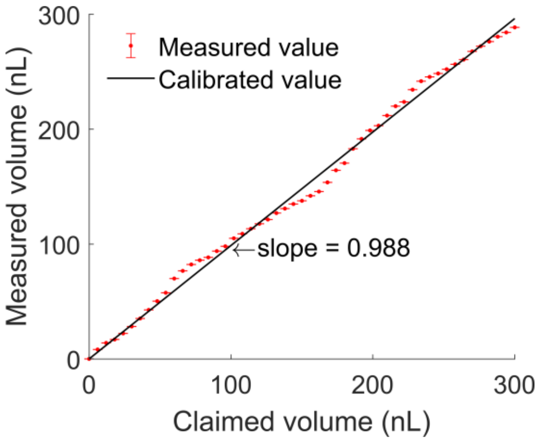

Fig. S6 Calibration of dispensed liquid volume. A series of liquid volumes claimed by the dispenser was dispensed on the glass substrate to form the liquid lens, and the volume was measured (with a measurement accuracy of ± 0.05 nL). The slope of the fitted line is about 0.988 and is used to calibrate the claimed dispensed volume.

Figure S7. Refractive index profile of NOA 81 under room temperature.

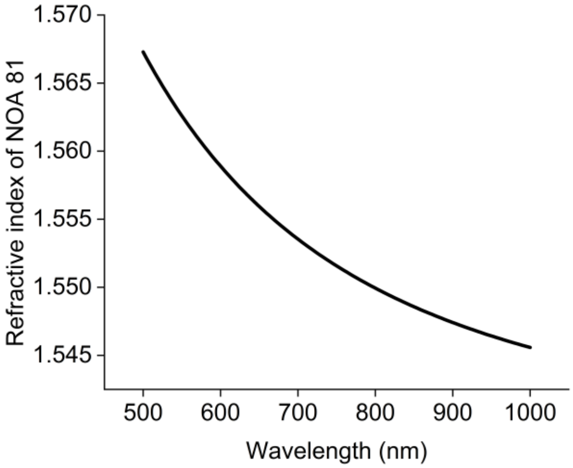

Fig. S7 Refractive index profile of NOA 81 under room temperature.

Figure S8. Schematic of mouse brain handling for histology.

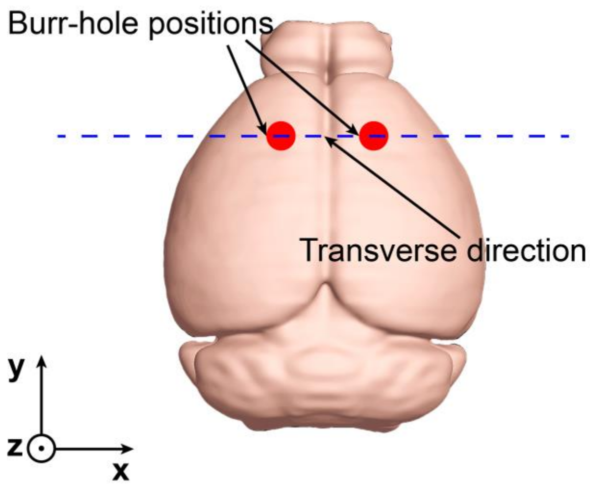

Fig. S8 Schematic of mouse brain handling for histology. The red dots indicate the burr-hole positions where the glass capillary tubes were inserted. The blue dashed line indicates the transverse direction for brain tissue dissection (the cross-section parallel to the x-z plane).

Supplementary Table 1: Volume variation of microlenses before and after polymerization.

Volume before exposure (V_1, nL)	Volume after exposure (V_2, nL)	Shrinkage ratio ($\frac{V_1-V_2}{V_1}$)	Averaged shrinkage ratio
1841.4	1712.0	7.03%	7.69%±0.39%
1521.7	1393.5	8.43%	
1263.7	1176.0	6.93%	
765.6	712.2	6.93%	
658.5	607.6	7.73%	
470.7	430.1	8.62%	
266.5	244.9	8.12%	
213.3	197.3	7.51%	
149.7	138.1	7.74%	
51.1	46.8	8.41%	
28.6	26.4	7.34%	
10.8	10.0	7.41%	

Supplementary Table 2: Characterization of the simultaneously fabricated liquid-shaped microendoscopes.

Characterized parameter	# 1*	# 2	# 3**	# 4	# 5
x focused spot size	4.5 μm	4.6 μm	4.5 μm	4.7 μm	4.5 μm
y focused spot size	4.3 μm	4.4 μm	4.3 μm	4.5 μm	4.2 μm
Mean focused spot size [a]	4.5 μm	4.6 μm	4.5 μm	4.7 μm	4.4 μm
Astigmatism ratio	1.05	1.05	1.05	1.04	1.07
Effective DOF	200 μm	202 μm	198 μm	204 μm	196 μm
Working distance	240 μm	235 μm	241 μm	237 μm	243 μm
Mean axial resolution [b]	2.43 μm	2.52 μm	2.43 μm	2.45 μm	2.51 μm
Axial resolution variation [c]	4.6%	4.7%	4.6%	5.0%	4.8%
Mean spectral variation [d]	0.0136	0.0141	0.0138	0.1315	0.0145

* The reported rigid microendoscope.

** The reported flexible microendoscope.

[a] Mean focused spot size = 0.83114 × x + 0.16886 × y when x ≥ y, mean focused spot size = 0.16886 × x + 0.83114 × y when x < y, and x and y indicate the beam diameters in x and y directions, respectively (provided by DataRay Inc.).

[b - c] The axial resolution is measured every 100 μm along the imaging depth of 100 μm to 1000 μm, and mean axial resolution and axial resolution variation can be calculated.

[d] The mean spectral variation (MSV) is calculated by MSV = MEAN(STD(S1, S2, …, Si)), where Si is a 2048 × 1 vector that contains the normalized spectral data, MEAN is the averaging operation, and STD indicates the standard deviation.

Supplementary Table 3: Comparison of fabrication methods for OCT microendoscopes.

Methods	Pros	Cons
Liquid shaping-based method	1. Enable monolithic design for minimization of OCT probe.<br>2. Freeform lens of high design freedom and rotational symmetric surface for aberration corrections.<br>3. Mass production of freeform lens.<br>4. Freeform lens of sub-nanometer surface roughness.<br>5. No need for lens polishing and precise optical alignment.<br>6. Scalable fabrication.<br>7. Short endoscope fabrication time of at most 1.5 hours.<br>8. Fabrication material cost of about $70, including single-mode fiber, non-core fiber, optical liquid, glass substrate, wetting materials, toque coil, and protective sheath.	1. Need a relatively expensive liquid dispensing system of about $30,000 or a home-made liquid dispensing system of about $6,000.
Two-photon 3D microprinting-based method	1. Enable monolithic design for minimization of OCT probe.<br>2. No need for lens polishing and precise optical alignment.<br>3. Freeform lens of high design freedom and complex asymmetric surface for aberration corrections.	1. Suboptimal surface roughness.<br>2. Lack of scalability potential.<br>3. Long printing time up to several hours, depending on the lens size.<br>4. Expensive two-photon printing machine, ownership cost of at least $500,000.<br>5. Limited choice of photo resins.
GRIN fiber-based method	Enable monolithic design for minimization of OCT probe and GRIN fibers are commercially available.	1. Limited choice of GRIN fibers and generally unknown fiber parameters.<br>2. Lack of capability to correct imaging aberrations.<br>3. GRIN fibers usually have strong chromatic and spherical aberrations at short wavelength regimes, such as 800 nm.<br>4. Requires costly and time-consuming angle-polishing.<br>5. Suboptimal surface roughness due to polishing.<br>6. Endoscope fabrication time up to several hours.
Fiber ball-lens-based method	1. Enable monolithic design for minimization of OCT probe<br>2. Provide achromatic performance at short wavelength ranges, such as 800 nm.	1. Insufficient design freedom and controllability on ball-lens using fiber melting technique.<br>2. Requires costly and time-consuming angle-polishing.<br>3. Suboptimal surface roughness due to polishing.<br>4. Limited to fabricate achromatic endoscopes of less than 1 mm in diameter.<br>5. Endoscope fabrication time up to several hours.

Supplementary Table 4: Six groups of glass substrates with different parameters of surface wettability modification.

Glass substrate group	Processing time [a]	Volume of fluoride [b]	Contact angle
Glass substrate 1	0 hour	0 μL	~ 50°
Glass substrate 2	4 hours	10 μL	~ 67°
Glass substrate 3	6 hours	10 μL	~ 75°
Glass substrate 4	8 hours	10 μL	~ 85°
Glass substrate 5	12 hours	10 μL	~ 90°
Glass substrate 6	16 hours	20 μL	~ 110°

[a] The processing time indicates the period when the sample is soaked in the fluoride solution.

[b] The fluoride is 1H,1H,2H,2H-Perfluorooctyltriethoxysilane used for modifying the wettability of the glass substrate.

Supplementary Table 5: Two groups of cylinder substrates with different parameters of surface wettability modification.

Cylinder substrate group	Processing time [a]	Volume of fluoride [b]	Contact angle [c]
Circular cylinder substrate	4 hours	10 μL	~65°
Elliptical cylinder substrate	8 hours	10 μL	~80°

[a] The processing time indicates the period when the sample is baked in a thermotank for fluorination.

[b] The fluoride is 1H,1H,2H,2H-Perfluorooctyltriethoxysilane used for modifying the wettability of the cylinder substrate.

[c] The contact angle is measured when the microlens is not constrained by the physical boundary.

Supplementary Table 6: Comparison between optical liquids.

Optical liquid	Cure Time [a]	Working Temperature [b]	Viscosity @ 25 °C [c]	Elastic Modulus [d]	Transmission @ VIS to NIR [e]
NOA60	25 minutes	-15~90°C	300 cps	135000 psi	96%
NOA61	25 minutes	-150~125°C	300 cps	150000 psi	96%
NOA63	40 minutes	-15~90°C	2000 cps	240000 psi	98%
NOA65	40 minutes	-15~60°C	1200 cps	20000 psi	98%
NOA68	40 minutes	-80~90°C	5000 cps	20000 psi	98%
NOA81	20 minutes	-150~125°C	300 cps	200000 psi	96%

[a] – [e] The data is obtained from Norland Product Inc.

Movie S1: Microlens on a circular cylinder substrate.

Movie S2: Microlens on an elliptical cylinder substrate.
