# Supplementary material for: Liquid-shaped microlens for scalable production of ultrahigh-resolution optical coherence tomography microendoscope
Source: Commun Eng. 2024 Jan 2;3:1. doi: 10.1038/s44172-023-00157-1 (PMC10955823; doi:10.1038/s44172-023-00157-1)
Supplement: Supplementary file 3 — Description of Additional Supplementary Files [file 44172_2023_157_MOESM3_ESM.pdf]

Description of Additional Supplementary Files

File name: Supplementary Movie 1

Description: Micro lens on a circular cylinder substrate.

File name: Supplementary Movie 2

Description: Micro lens on an elliptical cylinder substrate.
